# *In-silico* molecular analysis and blocking of the viral G protein of Nipah virus interacting with ephrin B2 and B3 receptor by using peptide mass fingerprinting

**DOI:** 10.3389/fbinf.2025.1526566

**Published:** 2025-06-25

**Authors:** Ayesha Sajjad, Ihteshamul Haq, Rabia Syed, Faheem Anwar, Muhammad Hamza, Muhammad Musharaf, Tehmina Kiani, Faisal Nouroz

**Affiliations:** ^1^ Department of Bioinformatics, Govt. Postgraduate College Mandian Abbottabad, Abbotabad, Khyber Pakhtunkhwa, Pakistan; ^2^ College of Life Sciences and Technology, Beijing University of Chemical Technology, Beijing, China; ^3^ Academy of medical engineering and translational medicine Tianjin University China, Tianjin, China; ^4^ Department of Bioinformatics, Hazara University Mansehra, Dhodial, Khyber Pakhtunkhwa, Pakistan

**Keywords:** peptide mass fingerprinting, paramyxovirus, ephrin B2 and B3 receptor, viral g protein, nipah virus (NiV)

## Abstract

**Introduction:**

The Nipah virus (NiV), a zoonotic paramyxovirus closely related to the Hendra virus, poses a significant global health threat due to its high mortality rate, zoonotic nature, and recurring outbreaks primarily in Malaysia, Bangladesh, and India. Infection with NiV leads to severe encephalitis and carries a case fatality rate ranging from 40% to 75%. The lack of a vaccine and limited understanding of NiV pathogenesis underscore the urgent need for effective therapeutics. This study focuses on identifying viral peptides of the Nipah virus using the peptide mass fingerprinting technique. This approach identified antiviral peptides acting as potent inhibitors, targeting the viral G-protein’s interaction with cellular ephrin-B2 and B3 receptors. These receptors are crucial for viral entry into host cells and subsequent pathogenesis.

**Methods:**

Identifying NiV viral peptides not only enhances our understanding of the virus’s structural and functional properties but also opens avenues for developing novel therapeutic strategies. By blocking the interaction between the viral G-protein and host receptors, these antiviral peptides offer promising prospects for drug development against NiV.

**Results and Discussion:**

Twenty-one peptides were identified using peptide mass fingerprinting. These peptides were then subjected to docking analysis with two antiviral peptides of the ephrin B2 receptor and a monoclonal antibody, demonstrating robust stability and binding affinity. These predicted peptides contribute to the broader field of virology by elucidating key aspects of NiV biology and paving the way for the development of targeted antiviral therapies. Future studies may further explore the therapeutic potential of these peptides and their application in combating other viral infections.

## Introduction

Since the COVID-19 pandemic, more time and resources have been allocated to investigate the pandemic, such as the Nipah virus (T. Johnson et al., 2023; Liew et al., 2022). The Nipah virus, referred to as NiV, a novel zoonotic paramyxovirus closely related to the Hendra virus, was just recently discovered by scientists 23 years ago (Gazal et al., 2022; Gurley et al., 2020). It is a pleomorphic virus that is a member of the henipavirus genus and the *Paramyxoviridae* family (Dawes and Freiberg, 2019; Liew et al., 2022; Marsh and Netter, 2018). The initial isolates of the virus were discovered at Sungai Nipah (Nipah River Village), hence named the “Nipah virus” (Angeletti et al., 2016; Ochani et al., 2019).

Following that, studies revealed that pteropid bats (*Chiroptera: Pteropodidae*) are the natural reservoir of NiV (DeBuysscher et al., 2021; Epstein et al., 2020; Mougari et al., 2022). In the Malaysian town of Kampung Sungai Nipah, the first known human infection of the Nipah virus occurred in 1998, sparking a fatal outbreak that persisted until 1999 (Chauhan et al., 2018; Hauser et al., 2021; Talukdar et al., 2023). Pigs that were NiV-infected—the virus’s intermediate hosts—were the source of human infection in Malaysia. Human-to-human infection may occur through direct touch, aerosols, or fomites, while viral transmission from pigs to humans happens through direct contact with suffering pigs (Arunkumar et al., 2019; Bruno et al., 2022; Kummer and Kranz, 2022; Verma et al., 2018). Following the process of virus isolation and sequencing, the perpetrator was identified as a new RNA virus belonging to the *Paramyxoviridae* family, which includes the measles (MeV), mumps (MuV), and Hendra (HeV) variants (Goh et al., 2020; Lo Presti et al., 2016; Sun et al., 2018; Weingartl, 2015). Since then, smaller, more irregular outbreaks have occurred almost yearly throughout South Asia, with case fatality rates rising beyond 90% (Hauser et al., 2021; Nikolay et al., 2019; Spiropoulou, 2019). Almost every year, from 2001 to 2013, there were many human Nipah cases in Bangladesh, with a fatality rate of 70% (Kulkarni et al., 2013; 2017). With outbreaks found in Kerala, India, in 2018 and the Philippines in 2014, the geographic range of human cases of Nipah virus infection has continued to expand (Arunkumar et al., 2019; Ching et al., 2015; Gurley et al., 2020).

Both humans and animals are susceptible to a wide range of diseases from NiV, including moderate to severe encephalitis or fatal respiratory illnesses (Sharma et al., 2019; Talukdar et al., 2023). The infection frequently causes fevers that initially appear flu-like and are followed by irritations, comas, and ultimately death (Goh et al., 2020; Lo Presti et al., 2016). The NiV virus has been acknowledged by the World Health Organization (WHO) as a global health concern because of its high human mortality rate, (Skowron et al., 2022).

Similar to other paramyxoviruses in terms of morphology, NiV is an enclosed virus that is pleomorphic, spherical, or thread-like, measuring between 40 and 1,900 nm in diameter and having a single layer of surface protrusions that are typically 17 nm in length (Ang et al., 2018; Sharma et al., 2019; Skowron et al., 2022). The single-stranded, non-segmented, negative-sense RNA genome of paramyxoviruses is completely enclosed by envelope proteins, which include a distinct fusion (F) protein and a cell receptor binding protein known as glycoprotein (G) of henipaviruses, hemagglutinin (H), or hemagglutinin/neuraminidase (HN) (Aguilar et al., 2016; Hauser et al., 2021). The 18.6 kb negative-sense single-stranded RNA (ssRNA) genome of NiV has 6 genes that code for 9 different proteins: (Chaudhary et al., 2023; Goh et al., 2020; Gupta et al., 2020): three non-structural and six structural. The three non-structural proteins—C, V, and W—are essential to the pathophysiology of NiV because they control the early host pro-inflammatory response and the emergence of respiratory symptoms. The six primary structural proteins are large protein (L), phosphoprotein (P), matrix protein (M), nucleocapsid (N), fusion protein (F), and glycoprotein (G) (Ang et al., 2018; Hassan et al., 2022; Satterfield et al., 2015). The F and G proteins which cover the envelope regulate attachment and entrance into the host cell. While the F protein causes viral-cell membrane fusion, which makes it easier for the virion to enter the host, the G protein promotes virus attachment and attaches to the host’s cells Ephrin-B2 and -B3 receptors (Hauser et al., 2021; Singh et al., 2019). NiV takes four to 21 days to incubate (Aditi and Shariff, 2019; Saha et al., 2024).

Numerous vital organs, including the brain, lungs, heart, kidneys, and spleen, can be impacted by a Nipah virus infection (Skowron et al., 2022; Thakur and Bailey, 2019). Patients infected with NiV may appear clinically with a variety of symptoms, including asymptomatic infections, coughing with respiratory distress, encephalitis, or meningitis (Banerjee et al., 2019; Kenmoe et al., 2019; Mukherjee et al., 2018). To infect humans and other animals, NiV penetrates through the oronasal pathway (Clayton et al., 2016; Liew et al., 2022). It has been shown by several recent studies conducted on animal models that the virus could enter the central nervous system through the olfactory nerve instantly and then pass into the cerebrum through the choroid plexus, a network of blood veins in the cerebrum. This infection can cause disruptions to the blood-brain barrier (BBB), which can result in some neurological issues (Talukdar et al., 2023; Tiong et al., 2018). The attachment of the viral G protein to the cellular receptors ephrin-B2 and -B3 initiates the NiV infection of host cells (Aktaş et al., 2023; Liew et al., 2022; Tritsch, 2023). Confirmation of the presence of infections in humans and animals requires virus isolation, serological testing (an antibody test that searches the blood for antibodies), and assays for viral nucleic acid amplification (Garbuglia et al., 2023; Mazzola and Kelly-Cirino, 2019; Talukdar et al., 2023).

PMF is a high-throughput protein identification technique that permits the identification of a protein by fusing MS data with search techniques on an appropriate protein database as long as the protein’s amino acid sequence is known and recorded in the protein database (Tiengo et al., 2009; Vitorino et al., 2020). In peptide mass fingerprinting, an unknown protein is broken down by endoprotease typically trypsin to produce the individual minute peptides (Saraswathy and Ramalingam, 2011). The PMF method involves cleaving proteins at specific sites using enzymes like trypsin, which is derived from the pancreas. Trypsin selectively breaks peptide bonds at the C-terminal ends of lysine (Lys, K) and arginine (Arg, R) residues, unless followed by proline. This enzymatic digestion yields a series of peptides known as tryptic peptides, each ending with either lysine or arginine.(García-Vázquez et al., 2023). Subsequently, the exact mass of the peptides is determined using mass spectroscopy analysis, which provides a peak inventory catalog of the detected peptides, After this peak list is compared to the theoretical peptide peak list derived from the *in silico* digestion of the database proteins (Hamza et al., 2021; Saraswathy and Ramalingam, 2011).

This methodological approach not only aids in deciphering the intricate composition of proteins but also plays a pivotal role in advancing our understanding of their biological functions and interactions. These peptides possess characteristic masses corresponding to their amino acid sequences, facilitating identification based on mass spectrometry analysis. In essence, PMF provides insights into protein sequence variations by analyzing the unique mass profiles of peptides derived from enzymatic digestion, thereby enabling precise identification of proteins in biological samples. (García-Vázquez et al., 2023). The core principle of PMF relies on the comparison of these experimental mass spectra with theoretical peptide masses generated through computational algorithms utilizing comprehensive protein databases. This comparison enables the identification of 33 proteins by matching the observed mass fingerprints with the most compatible entries in the database, thereby pinpointing the protein’s identity based on its distinct peptide profile. (Hamza et al., 2021).

The benefit of Peptide mass fingerprinting is that it's a comparatively simple method that may be applied to low-cost, high-throughput applications (Buckley, 2016; Kaur et al., 2019). Analytical proteomics benefits greatly from peptide mass fingerprinting because it combines a conceptually straightforward method with reliable, high-throughput instrumentation (Dodds et al., 2006) PMF is essential for detecting proteins that are difficult to identify using other techniques because it can detect proteins with high sensitivity, even those with low abundance. The capacity to filter out non-peptide signals increases this sensitivity, boosting the accuracy and discrimination of protein identifications (Doran et al., 2007) For extensive proteome research, the method’s conceptual simplicity and automation make it advantageous. It uses strong search algorithms and reliable equipment, like MALDI-TOF MS, to automate the identification procedure (Damodaran et al., 2007).

*In-silico* peptide mass fingerprinting was used to find the peptides with anti-cancer properties. Finding the anti-cancerous peptides from the medicinal plant Calotropis gigantea is the main goal of this investigation. The active peptides from a species are discovered using peptide mass fingerprinting, which compares them to the already recognized peptides of other species in the database. (Rehman et al., 2021). in another study the Peptide Mass Fingerprinting Technique (PMFT) was used to identify the 55 A. baumannii strains that were isolated from 220 specimens of various animal flesh. The qPCR approach was used to genotype all detected isolates for the presence of genes linked to biofilms (ompA, bap, blaPER-1, csuE, csgA, and fimH). PMF is a robust, quick method that can identify roughly 97% of all isolates at the species level. (Elbehiry et al., 2021). Also the viral peptides of SARS-CoV-2 were identified using peptide mass fingerprinting. Following the identification of fifteen viral peptides for which 3D structures were predicted, three compounds—hydroxychloroquine, kaempferol, and anthraquinone—were selected for this investigation based on their antiviral qualities, which included the ability to bind to the target and nontoxicity. (Hamza et al., 2021). In this study, the peptide mass fingerprinting technique is used to identify viral peptides of the Nipah virus and identify anti-viral peptides that act as potent inhibitors against the Nipah virus. These antiviral peptides will prevent the viral G-protein from interacting with the cellular ephrin-B2 and -B3 receptors.

Gap: The absence of FDA-approved vaccines or drugs for Nipah virus infection propounds the virus’s situation as a serious public health concern. Although there have been a few studies on promising therapeutic approaches, the research on specific antiviral strategies aimed at blocking the interaction of the G-protein of Nipah virus with its host receptors is still missing. (Skowron et al., 2022).

We identified viral peptides of Nipah virus using peptide mass fingerprinting and selected two antiviral peptides targeting these viral peptides—one from the ephrin-B2 receptor and another from a monoclonal antibody. Then, docking studies were performed, which led to a promising result of our work. This study also attempts to narrow the gap in possible antiviral peptide-based treatment by providing stronger computational evidence for their role in disrupting the virus’s infection of host cells. Our findings could lay down a basis that may be further validated experimentally to try in the development of effective therapeutic approaches against Nipah virus.

## Materials and methods

**FIGURE 1:**
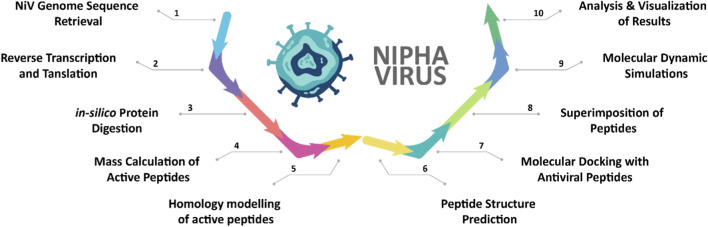
Schematic representation of overall methodology

The comprehensive computational workflow employed in this study to analyze the Nipah virus (NiV) proteome and identify potential antiviral peptides is shown in Figure 1.

### NiV genome sequence retrieval

The full genome sequence of Nipah was available from the NCBI (https://www.ncbi.nlm.nih.gov/), with accession number NC002728.1, 18250 bp long and known to cause encephalitis and respiratory illnesses. It was first isolated in the Malaysian town of “Sungai Nipah.”(Sah et al., 2024).

### Reverse transcription and translation of NiV sequence

As the Nipah (NiV) virus is a non-segmented negative-sense RNA virus, it needs reverse transcription to proliferate. Thus, reverse complement was used to execute reverse transcription (https://www.bioinformatics.org/sms/rev_comp.html) (2). EMBL EMBOSS TRANSEQ is used for translation (https://www.ebi.ac.uk/jdispatcher/st/emboss_transeq). It enabled the process of translating the nucleotide sequence into amino acids. Six reading frames were available: three in the forward direction and three in the reverse. Moreover, this could yield multiple outputs simultaneously. (Rehman et al., 2021; Hamza et al., 2021).

### *In-silico* protein digestion

The protein sequence was digested by Protein Prospector’s MS digest https://prospector.ucsf.edu/prospector/cgi-bin/msform.cgi?form=msdigest) A protein sequence can be entered to perform a single *in silico* digestion. All aspects of cleavage sites, digested peptide sequences, and sequence coverage can be computed and exhibited by iterative manual analysis to determine the best possible combination of digestion strategies (Lu et al., 2008). Trypsin is most frequently used to carry out proteolysis (Hoofnagle et al., 2016).

### Mass calculation of active peptides

The Peptide Mass Tool was used to calculate the mass of the peptide (https://web.expasy.org/cgi-bin/peptide_mass/peptide-mass.pl). The PeptideMass tool aims to assist in peptide mapping studies, as well as the interpretation of data from mass spectrometry and peptide-mass fingerprinting (PMF) results (Gasteiger et al., 2005).

### Homology Modelling of active peptides

After obtaining the peptide fragments from Protein Prospector, Mascot (https://www.matrixscience.com/cgi/search_form.pl?FORMVER=2&SEARCH=PMF) search analysis was performed for homology modeling. Mascot matches theoretical peptide sequences obtained from protein databases with experimental mass spectrometry data to identify peptides. (Sharma et al., 2018).

### Peptide structure prediction

The PEPFOLD3 (https://mobyle.rpbs.univ-paris-diderot.fr/cgi-bin/portal.py#forms::PEP-FOLD3) server was utilized to predict peptide structures. This server allows for the *de novo* structure prediction of linear peptides ranging from 5 to 50 amino acids, as well as the creation of native-like conformities of peptides interacting with proteins when the interaction site is recognized (Lamiable et al., 2016).

### Binding of active peptides with antiviral peptides

To investigate the effectiveness against the Nipah virus, the two antiviral activity-containing peptides were docked with the specified active peptides of the Nipah virus. The ephrin-B2 receptor contains the peptide **FSPNLW**, which interacts with the G protein. The monoclonal antibody includes the peptide **LAPHPSQ.** In their 2019 study, Sen et al. used these peptides as inhibitors of the G proteins because they bind to the ephrin receptor binding pocket, which stops the virus from attaching to the host cell. (Sen et al., 2019).

Molecular docking studies were performed with ClusPro (https://cluspro.org) to determine interactions between these two antiviral peptides and predicted peptides. One of the most popular docking servers, ClusPro, enables the prediction of protein-protein interactions by generating binding conformations based on energy minimization and clustering algorithms and ranking them according to their energies. One protein, referred to as the receptor, is positioned on a fixed grid at the coordinate system’s origin, while the other protein, referred to as the ligand, is positioned on a movable grid. The interaction energy is expressed as a correlation function (or as the sum of several correlation functions).(Kozakov et al., 2017).

Cluspro performs protein-protein docking using three main parameters.

• Sampling billions of conformations for rigid body docking
• Grouping of the 1,000 lowest-energy structures produced using root-mean-square deviation (RMSD) to identify the largest clusters that will serve as the most realistic models of the complex;
• Optimization of certain structures through energy minimization (Vajda et al., 2017)

### Analysis and visualization of results

The resulting docked complexes were examined and visualized using PyMOL and BIOVIA Discovery Studio to assess and compare the precise binding of various antiviral compounds, offering a three-dimensional (3D) platform for result visualization. PyMOL and BIOVIA Discovery Studio display the sizes and positions of binding sites, hydrogen bond interactions, hydrophobic interactions, and bonding distances, showing interaction radii within <5 Å from the docked ligand’s position (Afriza et al., 2018).

## Results

The full 18 kb genome sequence of Nipah virus was obtained from the NCBI database. Due to its sufficient length, the sequence did not require fragmentation. Subsequently, this nucleotide sequence was subjected to peptide mass fingerprinting for further analysis. The sequence of the Nipah virus was translated into its corresponding amino acid sequence to achieve the intended objective and determine its peptide masses. The EMBOSS TRANSEQ server was employed for the translation of the Nipah genome sequence. From all potential translations generated, the complete amino acid sequence was selected for peptide mass calculations.

*In silico* peptide, mass calculations yielded peptide masses with precision up to 4–5 decimal places for all generated amino acid sequences following enzymatic digestion. Average mono-isotopic and isotopic mass values were also provided for these modified peptides. The Peptide Mass online server was utilized for the identification of peptide masses. The amino acid sequence of the Nipah virus was subjected to *in silico* trypsin digestion with selected online parameters. A threshold was set to exclude peptides with masses below 500 Da, as these might be too small for visualization in mass spectrometry. Additionally, allowance was made for one missed cleavage during digestion. Peptide masses were subsequently calculated, along with the mass values for the respective protein and theoretical isoelectric points.

Protein Prospector was employed for cross-validation purposes. The analysis covered 99.8% of the sequence in the obtained results as shown in Table 1.

**TABLE 1:** Peptide mass server calculations (average mass of protein/peptide, theoretical pI), total sequence coverage, missed cleavages (MC), and selected enzyme for sequence cleavage.

Protein Mass	Peptide masses range	pl	Total coverage	Missed cleavages (MC)	Enzyme
3926947.84	17476.2030 to 500.1996	9.19	99.8%	1	Trypsin

Because NiV infection is caused by a virus, the entire viral taxonomy was targeted in MASCOT with the enzyme trypsin while including one missed cleavage, so that up to 1 mutation among related peptides could be allowed; the peak list in this study was imported as a peptide mass fingerprinting data file and SwissProt was the selected search database. The six strongest matches were found in the homology search results with a p-value that was significantly less than 0.05 at most and the highest score up to 46 shown in Table 2.

**TABLE 2:** Identified matched proteins from the MASCOT server, including their accession numbers, masses, descriptions, p-values, and respective scores.

S.No	Accession No	Mass	Description	Threshold p	Score
1	Q9IH63.1	60243	Fusion glycoprotein F0 OS = Nipah virus	<0.05	46
2	Q6GZU0.1	22733	Uncharacterized protein 036L OS = Frog virus	<0.05	19
3	P33794.1	6845	Truncated 3-beta hydroxy-5-ene steroid dehydrogenase homolog OS = Variola virus	<0.05	9
4	P80605.1	1701	DNA-binding protein H3-RL (Fragment) OS = Rhizobium leguminosarum	<0.05	8
5	A9NEV1.1	7041	Large ribosomal subunit protein bL28 OS = Acholeplasma laidlawii	<0.05	9
6	Q5UPU2.1	29783	Uncharacterized protein R252 OS = Acanthamoeba polyphagia mimivirus	<0.05	14

After obtaining the peptide fragments from Protein Prospector, we used Mascot for homology modeling. Mascot works by comparing experimental mass spectrometry data to theoretical peptide sequences from protein databases. Through this process, the viruses and proteins containing peptide sequences that matched the experimental data were identified, along with their full annotations as shown in Table 3.

Since the 3D structure defines the function of each one, we have to predict the structures of identified peptides. As a result, Peptide structures were predicted for each matched peptide sequence using the *de novo* peptide prediction approach as shown in Table 4.

**TABLE 3:** Description of identified peptides from the MASCOT database, including accession numbers, protein names, expected versus calculated masses, and calculated pI values of the proteins/matched peptides.

S.No	Accession No	Protein name	Mr (expt)	Mr (calc)	pl (calc)	Matched peptides
1	Q9IH63.1	Fusion glycoprotein	3097.6087	3097.6087	5.84	CYCNLLILILMISECSVGILHYEKLSK
2	Q9IH63.1	Fusion glycoprotein	4094.1655	4094.1656	5.84	NNTHDLVGDVRLAGVIMAGVAIGIATAAQITAGVAL YEAMK
3	Q9IH63.1	Fusion glycoprotein	3641.8401	3642.9477	5.84	LAGVIMAGVAIGIATAAQITAGVALYEAMKNADNINK
4	Q9IH63.1	Fusion glycoprotein	3291.5988	3292.7442	5.84	LQETAEKTVYVLTALQDYINTNLVPTIDK
5	Q9IH63.1	Fusion glycoprotein	3635.8010	3635.8011	5.84	ELVVSSHVPRFALSNGVLFANCISVTCQCQTTGR
6	Q9IH63.1	Fusion glycoprotein	4299.9876	4301.1162	5.84	YLGSVNYNSEGIAIGPPVFTDKVDISSQISSMNQSLQ QSK
7	Q9IH63.1	Fusion glycoprotein	4979.7808	4979.7808	5.84	EAQRLLDTVNPSLISMLSMIILYVLSIASLCIGLITFISF IIVEK
8	Q6GZU0.1	Uncharacterized protein	3032.5700	3033.6070	9.46	MTLPDVSGSLGPLSPGTNGTLWAVGPRVVR
9	Q6GZU0.1	Uncharacterized protein	2400.2306	2400.3681	9.46	VVRYQIPALAYLTPGALWTLR
10	Q6GZU0.1	Uncharacterized protein	2302.2255	2303.2790	9.46	YQIPALAYLTPGALWTLRTR
11	Q6GZU0.1	Uncharacterized protein	3055.4280	3054.6040	9.46	DSIRTLHAVHYDVWTLGPLGPLGPTSPR
12	Q6GZU0.1	Uncharacterized protein	2166.1870	2167.0552	9.46	GPSARPCRLQTDSLHSTDAR
13	Q6GZU0.1	Uncharacterized protein	2384.1358	2385.2402	9.46	KDMSPFSFPGILEPSHLVGSLK
14	Q6GZU0.1	Uncharacterized protein	2596.4450	2597.3312	9.46	DMSPFSFPGILEPSHLVGSLKSPR
15	Q6GZU0.1	Uncharacterized protein	2715.3465	2716.3842	9.46	SPRVDPGVPCRPLALWGHPYQCLR
16	Q6GZU0.1	Uncharacterized protein	3037.5272	3038.4003	9.46	CLHPHCFPAAPGRPWDPWCRPDRLDP
17	P33794.1	Truncated dehydrogenase	2074.0274	2074.0921	4.72	MTVYAVTGGAEFLGRYIVK
18	P33794.1	Truncated dehydrogenase	1872.8916	1874.0513	4.72	YIVKLLISADDVQEIR
19	P33794.1	Truncated dehydrogenase	1534.7714	1535.8559	4.72	VINVVEDPQPLVSKVK
20	P33794.1	Truncated dehydrogenase	1916.9856	1918.0346	4.72	VKVINYIQCDINDLIR
21	P80605.1	DNA-binding protein	1458.7678	1459.7453	5.91	MNKNELVSAVAER

**TABLE 4:** Predicted peptide structures using the PEP-FOLD 3 server, including protein names, missed cleavages, and amino acid sequences of the respective structures.

S. No	Protein name	Peptides	Peptides structures
Peptide 1	Fusion glycoprotein	CYCNLLILILMISECSVGILHYEKLSK	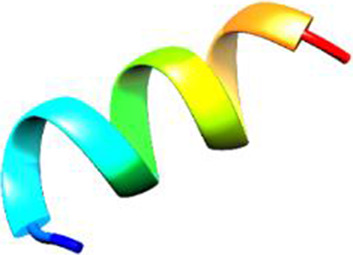
Peptide 2	Fusion glycoprotein	NNTHDLVGDVRLAGVIMAGVAIGIATAAQITAGVALYEAMK	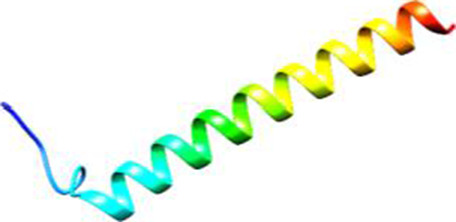
Peptide 3	Fusion glycoprotein	LAGVIMAGVAIGIATAAQITAGVALYEAMKNADNINK	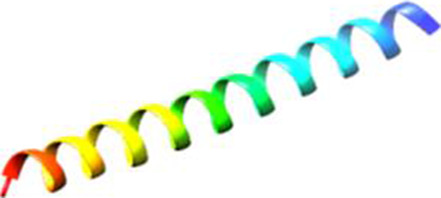
Peptide 4	Fusion glycoprotein	LQETAEKTVYVLTALQDYINTNLVPTIDK	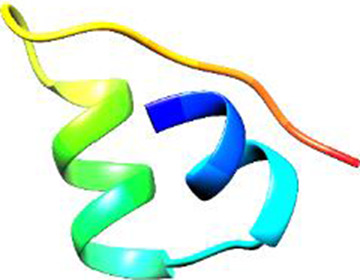
Peptide 5	Fusion glycoprotein	ELVVSSHVPRFALSNGVLFANCISVTCQCQTTGR	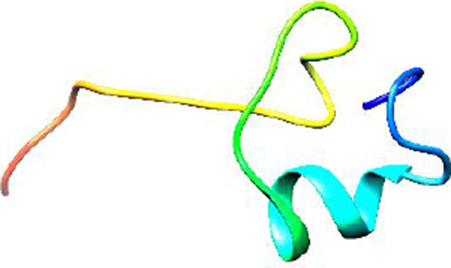
Peptide 6	Fusion glycoprotein	YLGSVNYNSEGIAIGPPVFTDKVDISSQISSMNQSLQQSK	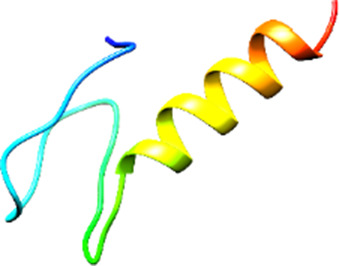
Peptide 7	Fusion glycoprotein	EAQRLLDTVNPSLISMLSMIILYVLSIASLCIGLITFISFIIVEK	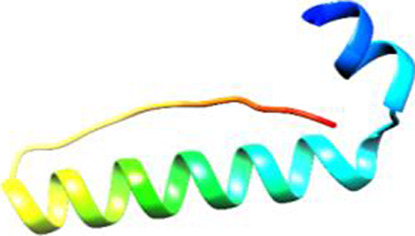
Peptide 8	Uncharacterized protein	MTLPDVSGSLGPLSPGTNGTLWAVGPRVVR	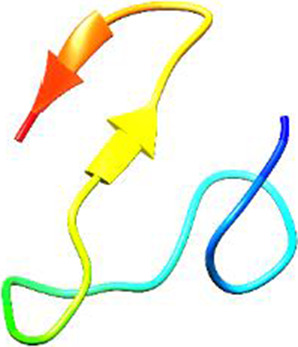
Peptide 9	Uncharacterized protein	VVRYQIPALAYLTPGALWTLR	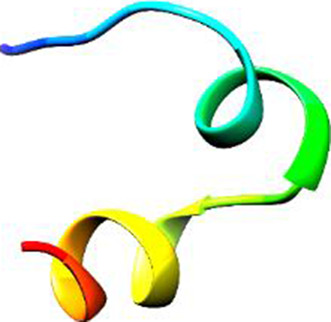
Peptide 10	Uncharacterized protein	YQIPALAYLTPGALWTLRTR	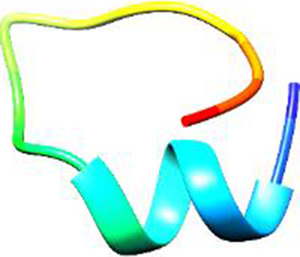
Peptide 11	Uncharacterized protein	DSIRTLHAVHYDVWTLGPLGPLGPTSPR	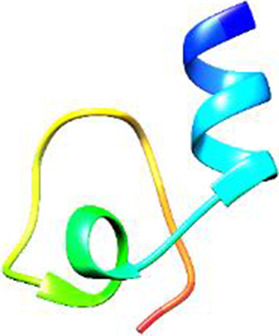
Peptide 12	Uncharacterized protein	GPSARPCRLQTDSLHSTDAR	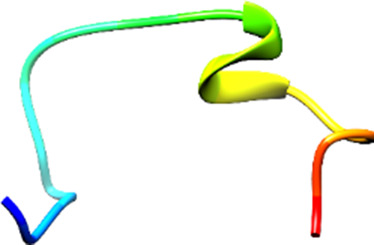
Peptide 13	Uncharacterized protein	KDMSPFSFPGILEPSHLVGSLK	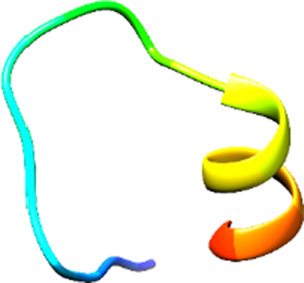
Peptide 14	Uncharacterized protein	DMSPFSFPGILEPSHLVGSLKSPR	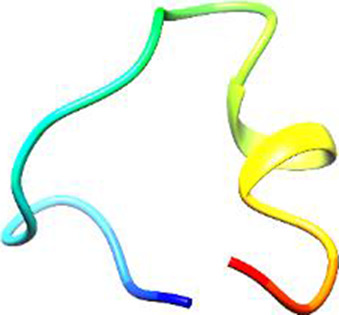
Peptide 15	Uncharacterized protein	SPRVDPGVPCRPLALWGHPYQCLR	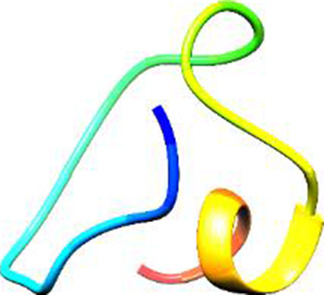
Peptide 16	Uncharacterized protein	CLHPHCFPAAPGRPWDPWCRPDRLDP	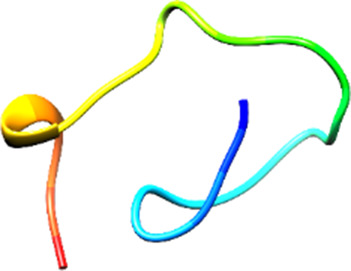
Peptide 17	Truncated dehydrogenase	MTVYAVTGGAEFLGRYIVK	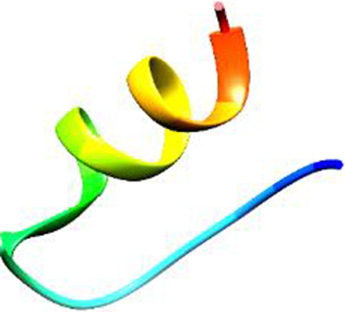
Peptide 18	Truncated dehydrogenase	YIVKLLISADDVQEIR	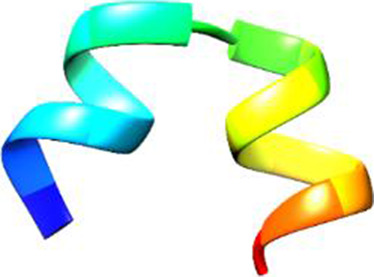
Peptide 19	Truncated dehydrogenase	VINVVEDPQPLVSKVK	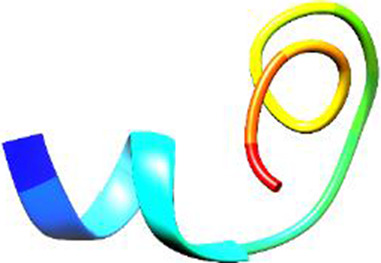
Peptide 20	Truncated dehydrogenase	VKVINYIQCDINDLIR	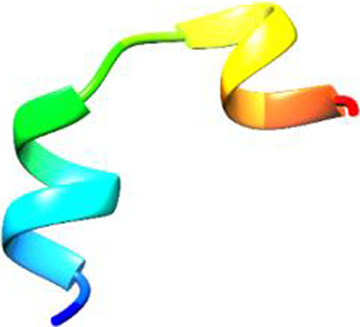
Peptide 21	DNA-binding protein	MNKNELVSAVAER	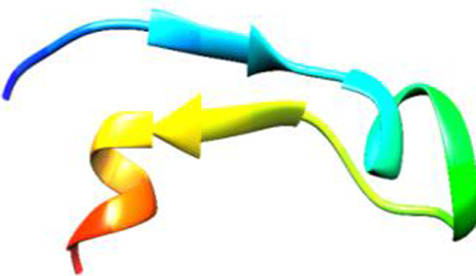

The FSPNLW peptide of the ephrin-B2 receptor and the LAPHPSQ peptide of the monoclonal antibody were attached to the identified NiV peptide structures. We bound the chosen antiviral peptides with the NiV peptides by using Cluspro. The Cluspro would require PDB files for the receptor and ligand, thus providing a list of poses ordered by kcal/mol that has the least amount of energy. The docking studies conducted using ClusPro for the ephrin-B2 receptor peptide (FSPNLW) revealed the lowest binding energies of −557.1 kcal/mol with 7 hydrogen bonds and −596.2 kcal/mol with 5 hydrogen bonds. Similarly, for the monoclonal antibody peptide (LAPHPSQ), the lowest binding energies recorded were −395.2 kcal/mol with 7 hydrogen bonds and −349.2 kcal/mol with 7 hydrogen bonds.

The Table 5 displays docked complexes listed from the most stable to the least stable based on binding energies obtained using ClusPro. Binding energy (kcal/mol) indicates the strength of interaction between the ligand and target protein; hence, the more negative the value, the stronger and more stable the interaction. The complex having the most stable conformation, with the lowest binding energy, is placed at the top of the table, representing strong binding affinity and likely effective interaction. Progressing downward through the table, the binding energies become less negative, indicating weaker interactions and lesser stability. While identifying the best potential inhibitors for further analysis, the complexes with higher stability are more inclined to establish strong viable bonds with the target protein at the risk of destabilizing interactions with other molecules.

**TABLE 5:** Binding energies of all docked complexes with both antiviral peptides as determined by ClusPro.

S. No	*FSPNLW peptide of Ephrine-B2 receptor*		Peptides	*LAPHPSQ peptide of Monoclonal antibody*	
Peptides	Lowest binding energies	Number of hydrogen bonds		Lowest binding energies	Number of hydrogen bonds
Peptide 6	−677.8	1H	Peptide 7	−483.1	1H
Peptide 4	−601.8	2H	Peptide 10	−452.5	2H
Peptide 9	−596.2	5H	Peptide 6	−445.4	0H
Peptide 3	−588.7	3H	Peptide 15	−444.7	1H
Peptide 14	−571.7	3H	Peptide 3	−437.6	4H
Peptide 11	−571.0	6H	Peptide 8	−431.8	3H
Peptide 5	−557.1	5H	Peptide 14	−427.5	1H
Peptide 15	−536.8	4H	Peptide 13	−418.3	1H
Peptide 10	−533.8	3H	Peptide 4	−409.9	6H
Peptide 7	−533.1	3H	Peptide 11	−395.2	7H
Peptide 13	−519.8	1H	Peptide 16	−391.4	3H
Peptide 8	−506.7	5H	Peptide 5	−386.4	6H
Peptide 16	−500.8	3H	Peptide 9	−380.2	5H
Peptide 1	−490.9	1H	Peptide 1	−377.1	4H
Peptide 12	−449.3	3H	Peptide 21	−371.3	4H
Peptide 2	−435.8	3H	Peptide 18	−355.0	0H
Peptide 21	−434.5	3H	Peptide 19	−349.2	7H
Peptide 18	−430.9	1H	Peptide 12	−340.6	5H
Peptide 19	−429.4	3H	Peptide 2	−322.4	1H
Peptide 20	−361.0	2H	Peptide 17	−316.4	1H
Peptide 17	−343.5	4H	Peptide 20	−295.5	2H

**FIGURE 2:**
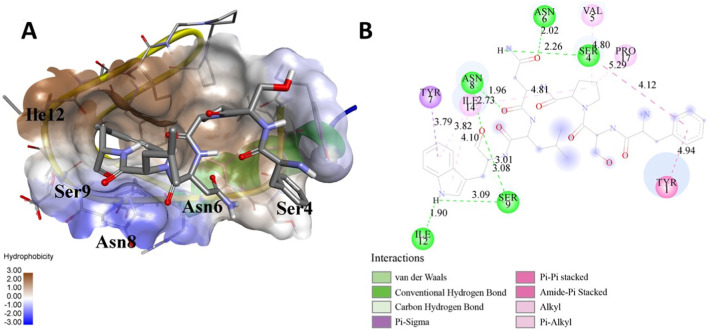
**(A)** The docked complex of the predicted peptide (ELVVSSHVPRFALSNGVLFANCISVTCQCQTTGR) with the ephrin-B2 receptor peptide (FSPNLW) demonstrates significant hydrogen bonding interactions with amino acid residues along with their positions, including Ille12, Ser9, Asn8, Asn6, and Ser4. The ligand peptide is observed to fit seamlessly into the hydrophobic surface of the receptor, achieving the lowest binding energy of −557.1 kcal/mol. **(B)** The 2D structure displays the amino acids that are acting with the peptide and their mode of interaction.

**FIGURE 3:**
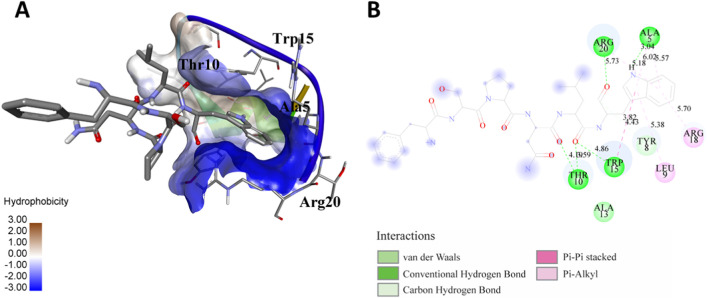
**(A)** The docking analysis of the predicted peptide (VVRYQIPALAYLTPGALWTLR) with the ephrin-B2 receptor peptide (FSPNLW) reveals critical hydrogen bonding interactions with Thr, Trp, Ala, and Arg residues at positions 10, 15, 5 and 20 respectively, showing how the ligand peptide fits into the hydrophobic pocket of the receptor, resulting in binding energy of −596.2 kcal/mol. **(B)** The 2D structural diagram displays the amino acid residues binding with the peptide, including the types of interactions.

**FIGURE 4:**
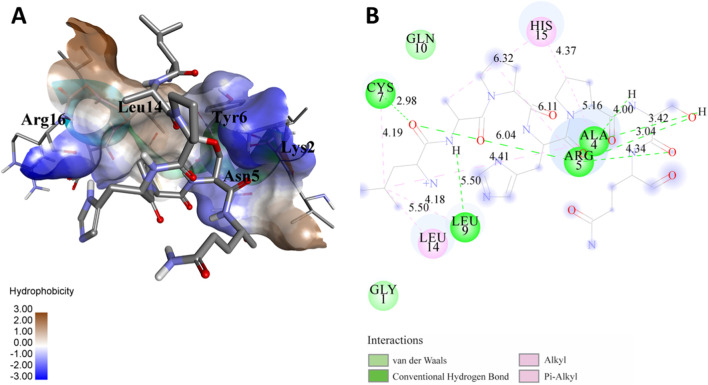
**(A)** The docking study of the predicted peptide (DSIRTLHAVHYDVWTLGPLGPLGPTSPR) with the monoclonal antibody peptide (FSPNLW) highlights substantial hydrogen bonding interactions along with the detailed information on the specific amino acid residues involved, and their positions are provided. The ligand peptide fits perfectly into the receptor’s hydrophobic surface, achieving a binding energy of −395.2 kcal/mol. **(B)** The accompanying 2D structure delineates the bound amino acids and their interactions.

**FIGURE 5:**
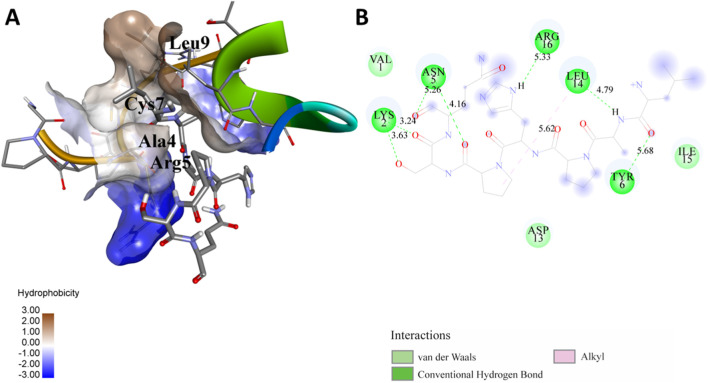
**(A)** The docking analysis of the predicted peptide (VINVVEDPQPLVSKVK) with the monoclonal antibody peptide (FSPNLW) reveals significant hydrogen bonding of Leu at position 9, Cys at 7, Ala at 4, and Arg at 5. Interactions. The ligand peptide is observed to fit seamlessly into the hydrophobic surface of the receptor, achieving a binding energy of −349.2 kcal/mol. **(B)** The corresponding 2D structural representation illustrates the amino acids interacting with the peptide, highlighting various types of interactions.

The following Figures 2–5 represent the docked complexes with the maximum number of hydrogen-bond interactions being formed. These interactions greatly stabilize and enhance the binding efficacy of such complexes. Being hydrogen-bonded in a larger number suggests stronger and favorable interactions between the peptide and the target protein, which validates the docking results.

**FIGURE 6:**
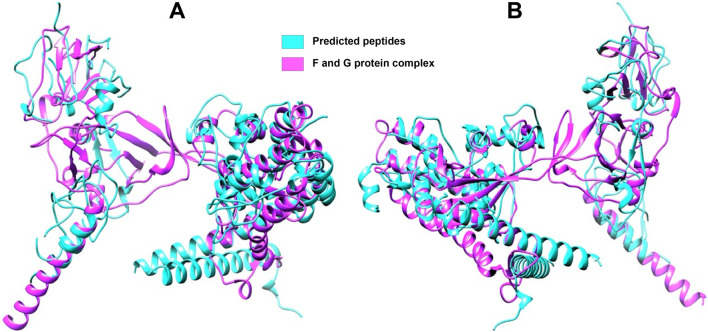
Full-length F and G protein complex with mapped peptides highlighted **(A)** Front side view **(B)** Rear side view. The magenta coloration delineates the overall structure of the F and G protein complex, which is crucial for viral entry and fusion with host cells. Predicted peptides, identified through *in silico* analysis, are marked in blue, highlighting regions of potential interaction and functional significance.

Figure 6 presents the mapping of predicted peptides onto the F and G protein complex of the Nipah virus, offering valuable insights into the structural and functional characteristics of these viral proteins. Through *in silico* analysis, predicted peptides are localized to specific regions on the complex, indicating potential interaction sites and areas of functional significance. The magenta coloration denotes the F and G protein complex, while the blue regions highlight the predicted peptides mapped onto this complex. This visualization aids in identifying key functional domains within the viral proteins that could be targeted for therapeutic intervention.

## Discussion

Nipah virus (NiV) is recognized as one of the deadliest viruses that infect human beings (Widerspick et al., 2022). Since its pathogenicity and potential for human-to-human transmission are high, NiV is considered a significant pathogen, and no approved treatments exist. (Johnson et al., 2021). The primary objective of this study is to predict viral peptides and identify small molecules that target these peptides. To attain this objective, peptide mass fingerprinting is employed for the identification of viral peptides. One of the main methods used for identifying peptides involves examining their molecular masses, known as Peptide Mass fingerprinting. It is the easiest and most efficient way to determine all peptides having certain masses, and it remains an essential tool in the advancement of proteomics (Hamza et al., 2021). Through a range of computational and bioinformatics analyses, twenty-one peptides were identified, derived from the Nipah virus genome. The three-dimensional structures of the accepted peptides were constructed and then employed in molecular docking studies with ClusPro. This was carried out to assess their binding affinities towards an ephrin-B2 receptor peptide as well as a monoclonal antibody peptide from existing literature (Sen et al., 2019). The docking outcomes with the lowest binding energies were compared with the corresponding literature to validate the results, showing an energy of −745.9 kJ/mol of Ephrin B2 receptor with complexes of NiV G protein (Hoque et al., 2023). The buoyancy in the predictions was essentially confirmed by the binding energies of the predicted peptides being found to be compatible with the published data.

To validate our peptides more, we also superimposed them on the complex structure of the F and G proteins of the Nipah virus (Talukdar et al., 2023). This was an important step as it helped confirm the spatial fit of our peptides on the viral proteins, hence guaranteeing that there would be effective binding on the appropriate portions of the G-protein and ephrin-B2/B3 receptors. (Hamza et al., 2021; Rehman et al., 2021). This superimposition also provides additional evidence toward the claim that our peptides can inhibit the Nipah virus G-protein from interacting with the Epherin-B2/B3 receptors. Our observations are consistent with what has already been reported in the literature concerning the structural studies of other proteins with the same functions. The G-protein interaction with the ephrin receptor was critical in the mechanism of entry of the Nipah virus (Priyadarsinee et al., 2022). Our research goes a step further by providing specific candidates of peptide clients with tested binding affinities, offering better insights into the structural interactions of the models.

The discovery of these twenty-one peptides allows for further investigations into the mechanisms of Nipah virus infection and host-cell interaction. These peptides bind to the important sites of viral G-protein and host ephrin-B2/B3 receptors that may be used to explore the druggability of those regions. In addition, the successful docking with monoclonal antibody peptides indicates that these peptides may also be useful in the design of antibody-based therapies (ProQuest, 2020). This study is a comprehensive computational analysis of these peptides. More experimental validation, including *in-vitro* and *in-vivo* studies, is important to establish the biological significance and binding ability of these peptides. Moreover, it would likely be beneficial to consider the possibility of peptide modifications, which could increase the stability and bioavailability of these peptides in terms of their use in therapy.

Viral resistance mechanisms, such as mutations and post-translational modifications in Nipah viruses (NiVs) and human immunodeficiency viruses (HIVs), can modify protein binding sites such that peptide inhibitors become less effective. These changes can modify inhibitor interaction and thus enable the virus to escape therapy. Such changes might meet other consequences, such as affecting peptide mass fingerprinting (PMF) by producing unexpected peptide fragments, altering cleavage patterns, and making identification more complicated. Consequently, resistant viral strains might go undetected or misidentified. This emphasizes the necessity for sophisticated analytical methods to address the issue of viral resistance and optimize inhibitor design (Sen et al., 2019).

One of the main limitations of PMF is that the protein concerned has to be characterized and cataloged in a reference database. This dependence results in a limited application, especially when analyzing novel or highly variable proteins that are being studied from emerging or less-studied organisms (Saraswathy and Ramalingam, 2011). The incorrect calibration of mass spectrometers is then very much critical for PMF as it introduces mass measurement errors and false identifications. PMF relies on the matching of experimentally determined peptide masses with those proteins in theory; thus, the minor deviations in calibration may lead to incorrect assignments of peptides. Miscalibration can result in false positives (incorrect matches with proteins associated with mass difference in the protein under consideration) or false negatives (not being able to identify the actual protein at all, possibly because the measured mass is outside of the error range defined). This is especially important in the detection of posttranslational modifications (PTMs) since changes in mass are very small and are usually masked by the instrument (Aebersold and Mann, 2003). During partial enzymatic digestion, the proteolytic enzyme might not cleave all target sites, resulting in peptides that contain one missed cleavage site. This failure to digest completely creates some odd peptide masses, thus complicating any database matching that occurs in peptide mass fingerprinting (PMF). As such, the accuracy of protein identification decreases, thus raising the chances of setting up false-positive identifications or, on the contrary, missed identifications (Siepen et al., 2007).

## Conclusion

This study provides considerable progress in investigating the Nipah virus (NiV) pathogenesis, whereby viral peptides are detected and characterized using advanced peptide mass fingerprinting. The entire sequence of the NiV genome was used to derive the protein sequences and calculate their corresponding masses to allow extensive searches in large databases that yielded twenty-one different viral peptides. Each of those peptides contributes to the understanding of the molecular structure of NiV, therefore making it possible to map its entire proteome. The central accomplishment of this research has to do with the structural estimations of these viral peptides and thus provides three-dimensional structural information that enhances the understanding of the functional activity of the Nipah virus (NiV) and assists in designing treatment strategies. Two, FSPNLW and LAPHPSQ antiviral peptides, were discussed in this study as possible candidates to disrupt the molecular machinery of NiV since they showed robust binding affinities, indicating good potential for use in therapy. The next imperative step entails the clinical translation of the observations made. There is a need for thorough examination and confirmation of these antiviral peptides in preclinical and clinical trials to evaluate their safety, efficiency, and the pharmacokinetics associated with their use. Improvement strategies may also be employed in a bid to increase their stability, bioavailability, and specificity towards NiV. In addition, there should be continuous research aimed at understanding the pathogenesis of NiV and utilizing such knowledge to formulate new ways of treatment. Potential means of addressing the need for antiviral medications have been uncovered using genomic, proteomic, and structural studies. Ongoing research and development efforts remain critical to ensuring that such knowledge will eventually be of benefit to public health anywhere in the world.

## Data Availability

The datasets presented in this study can be found in online repositories. The names of the repository/repositories and accession number(s) can be found in the article/supplementary material.
